# Postpartum infection, pain and experiences with care among women treated for postpartum hemorrhage in three African countries: A cohort study of women managed with and without condom-catheter uterine balloon tamponade

**DOI:** 10.1371/journal.pone.0245988

**Published:** 2021-02-08

**Authors:** Holly A. Anger, Jill Durocher, Rasha Dabash, Nevine Hassanein, Sam Ononge, Gillian Burkhardt, Laura J. Frye, Ayisha Diop, Seynabou Bop Moctar Beye Diop, Emad Darwish, Mohamed Cherine Ramadan, Juliana Kayaga, Dyanna Charles, Alioune Gaye, Melody Eckardt, Beverly Winikoff

**Affiliations:** 1 Gynuity Health Projects, New York, New York, United States of America; 2 Obstetrician/Gynecologist Consultant, Alexandria, Egypt; 3 Makerere University School of Health Sciences, Kampala, Uganda; 4 Department of Obstetrics and Gynecology, University of New Mexico, Albuquerque, New Mexico, United States of America; 5 Centre De Santé Philippe Senghor, Dakar, Senegal; 6 Alexandria Faculty of Medicine, Alexandria University, Alexandria, Egypt; 7 El Galaa Maternity Teaching Hospital, Cairo, Egypt; 8 Global Health Uganda, Kampala, Uganda; 9 Obstetrician/Gynecologist Consultant, Dakar, Senegal; 10 Global Health Innovation Lab, Massachusetts General Hospital, Boston, Massachusetts, United States of America; Guttmacher Institute, UNITED STATES

## Abstract

**Objective:**

We aimed to determine the risk of postpartum infection and increased pain associated with use of condom-catheter uterine balloon tamponade (UBT) among women diagnosed with postpartum hemorrhage (PPH) in three low- and middle-income countries (LMICs). We also sought women’s opinions on their overall experience of PPH care.

**Methods:**

This prospective cohort study compared women diagnosed with PPH who received and did not receive UBT (UBT group and no-UBT group, respectively) at 18 secondary level hospitals in Uganda, Egypt, and Senegal that participated in a stepped wedge, cluster-randomized trial assessing UBT introduction. Key outcomes were reported pain (on a scale 0–10) in the immediate postpartum period and receipt of antibiotics within four weeks postpartum (a proxy for postpartum infection). Outcomes related to satisfaction with care and aspects women liked most and least about PPH care were also reported.

**Results:**

Among women diagnosed with PPH, 58 were in the UBT group and 2188 in the no-UBT group. Self-reported, post-discharge antibiotic use within four weeks postpartum was similar in the UBT (3/58, 5.6%) and no-UBT groups (100/2188, 4.6%, risk ratio = 1.22, 95% confidence interval [CI]: 0.45–3.35). A high postpartum pain score of 8–10 was more common among women in the UBT group (17/46, 37.0%) than in the no-UBT group (360/1805, 19.9%, relative risk ratio = 3.64, 95% CI:1.30–10.16). Most women were satisfied with their care (1935/2325, 83.2%). When asked what they liked least about care, the most common responses were that medications (580/1511, 38.4%) and medical supplies (503/1511, 33.3%) were unavailable.

**Conclusion:**

UBT did not increase the risk of postpartum infection among this population. Women who receive UBT may experience higher degrees of pain compared to women who do not receive UBT. Women’s satisfaction with their care and stockouts of medications and other supplies deserve greater attention when introducing new technologies like UBT.

## Introduction

Postpartum hemorrhage (PPH) is the leading cause of maternal death worldwide and disproportionately affects women in low- and middle-income countries (LMICs) [1]. Health systems in LMICs face various challenges to addressing PPH, both in terms of providing timely access to interventions and providing overall high-quality care to women in what are often high-stress, under-staffed environments. While the rate of facility-based birth has increased in LMICs [2] and has afforded more women access to first-line PPH treatment such as uterotonics, a lack of blood supply and surgical capacity limits options for treating PPH that is not controlled by uterotonics [3, 4]. Uterine balloon tamponade (UBT) is an approach recommended by the WHO and the International Federation of Gynecologists and Obstetricians (FIGO) for the management of refractory PPH [5, 6] and can be provided using low-cost devices such as a condom tied to the end of a catheter, which is a feasible approach in low-resource settings [7]. Large, prospective case series conducted in LMICs report that bleeding is controlled for approximately 95% of women with refractory PPH who received condom-catheter UBT [8, 9]. However, results from two randomized controlled trials conducted in LMICs showed that condom-catheter UBT for refractory PPH did not result in lower rates of PPH-related invasive surgery or maternal deaths following vaginal birth [10, 11].

Unanswered questions remain around the safety and acceptability of UBT in LMICs, particularly regarding such factors as postpartum infection and pain. Most published data on safety and acceptability of UBT are from high-resource settings with conditions and practices that may contribute to more favorable outcomes. For example, insertion of the balloon is often done under anesthesia in the operating theater, the balloon is filled with sterile saline solution, and prophylactic antibiotics are given [12–14]. In contrast, potential UBT-related complications like infection and reported pain may differ in LMICs, where infection rates are generally higher after childbirth [15] and where pain management in childbirth is less frequently used [16, 17]. Published case series of UBT from LMICs report low rates of infection (~1.3%) following UBT use [8]; however, a recent secondary analysis of a large clinical trial conducted in several LMICs showed that UBT was associated with an increased risk of sepsis [18]. There is also limited published data from LMICs on pain reported by women who have UBT where anesthesia is less likely to be used or is unavailable, leading to a lack of evidence-based guidance on pain management for women who receive UBT.

While technologies like UBT may help address specific gaps in PPH care in LMICs, there is mounting evidence that strategies focused solely on expanding access to interventions may not result in reductions in maternal morbidity and mortality in these settings [19]. Indeed, improving the overall quality of care at facilities is indispensable to improving maternal and neonatal outcomes, and considering women’s experiences and values in the quality framework is an essential step to achieving high quality care [20, 21]. Research shows that women’s experiences with poor quality care and mistreatment at facilities in LMICs can be a barrier to use of health services and adherence to care recommendations [22–24]. Further, poor quality care and mistreatment can worsen the trauma experienced by women following a near-miss experience [25]. Understanding women’s experiences of care can help identify areas for quality improvement that could go hand-in-hand with introducing interventions like UBT that aim to improve maternal outcomes following PPH.

As part of a larger effectiveness trial of condom-catheter UBT introduction in three LMICs, we conducted a prospective cohort study to assess postpartum infection, pain, and care experiences reported by women diagnosed with PPH.

## Materials and methods

This cohort study was nested in a stepped wedge, cluster-randomized trial (i.e. effectiveness trial) of UBT introduction at 18 secondary level hospitals in Uganda, Egypt, and Senegal from October 2016 to March 2018 [11]. Ethical approval was obtained from the University of Alexandria Faculty of Medicine’s Research Ethics Committee (Alexandria, Egypt, Approved 25 May 2016), Makerere University Research Ethics Committee (Kampala, Uganda, Reference: SBS 366, Approved 16 June 2016), the Uganda National Council for Science and Technology (Kampala, Uganda, Reference: HS 3010, Approved 4 October 2016), and the National Council on Health Research, National Ethical Committee, Ministry of Health and Prevention (Dakar, Senegal, Reference: 00000120, Approved 11 August 2016). All participants in this cohort study provided written, informed consent.

This nested cohort study was designed to assess research questions around infection and pain associated with UBT use and around women’s general experiences with PPH care; these research questions are distinct from those related to effectiveness of UBT that were addressed in the overarching effectiveness trial. Briefly, the effectiveness trial assessed the impact of introducing UBT on the rate of PPH-related invasive surgery and maternal death among all vaginal deliveries at study sites. The intervention included a half-day training on use of condom-catheter UBT for refractory PPH. PPH diagnosis was defined as use of any interventions beyond prophylactic measures to control postpartum bleeding. If bleeding due to atonic PPH was not sufficiently controlled by uterotonics and other first-line interventions, providers were instructed to use UBT. The training recommended administering one dose of a broad-spectrum IV antibiotic for infection prophylaxis at the time of UBT insertion. The recommendations specified that an antibiotic was not needed if the woman had previously received antibiotics during labor for another reason. The training also advised providers not to delay placement of the balloon while awaiting antibiotics and that women should not stay on antibiotics unless needed for another indication. For management of pain during UBT placement, providers were instructed to give oral or IV analgesics, if needed, and provide reassurance to the woman during the procedure. Sites were provided pre-packaged kits containing locally procured components to assemble the condom-catheter balloon (i.e. Foley catheter, string, condoms, syringe, and catheter plug). Antibiotics and pain medications were not supplied by the study.

Only women diagnosed with PPH following vaginal birth were eligible for the nested cohort study and were approached after delivery and once they were in stable condition by trained staff who explained the study and administered written informed consent. Participation in the cohort study entailed a pre-discharge interview conducted in-person and a follow-up interview conducted in-person or via telephone approximately four weeks after delivery. While 6 weeks is the traditional time period used to monitor for postpartum endometritis [26, 27], the follow-up interview in this study was conducted at 4 weeks postpartum to minimize loss-to-follow-up since this time period was consistent with the local custom of a one-month rest period following birth when women and newborns are less likely to travel. Ethical approval for this research was obtained from the University of Alexandria Faculty of Medicine’s Research Ethics Committee (Alexandria, Egypt), School of Biomedical Sciences Research and Ethics Committee Makerere University College of Health Sciences (Kampala, Uganda), the Uganda National Council for Science and Technology (Kampala, Uganda), and the National Council on Health Research, National Ethical Committee, Ministry of Health and Prevention (Dakar, Senegal). Both the effectiveness trial and the cohort study are described on clinicaltrials.gov (NCT02910310).

Women enrolled in the nested study were divided into the following cohorts: women with PPH who received UBT (UBT group), and; women with PPH who did not receive UBT (no-UBT group). Women were eligible for the nested cohort study in time periods both before and after UBT was introduced as part of the effectiveness trial at the study sites. While most women in the UBT group of the nested cohort study were enrolled in the time period after UBT was introduced in the effectiveness trial, there were some women in the UBT group that were enrolled in the time period before UBT introduction due to several providers at study sites who were independently using UBT prior to its introduction as a component of the effectiveness trial. Information from interviews was documented on standardized data collection forms. Data on labor and delivery, PPH interventions, pain management, and administration of antibiotics during labor, delivery, and immediate postpartum were collected on data collection forms as part of the effectiveness trial and were also included in the analysis of the nested cohort study. The postpartum pre-discharge interview solicited information on women’s highest level of pain experienced postpartum (on a 0 to 10 scale) and pain experienced when the balloon was inserted (asked only to UBT cohort). The pre-discharge interview also solicited information about women’s overall experiences with care, if they called for help and why, if providers informed them that they had PPH, and if they were satisfied with their care and would recommend the facility to others. Five questions included a 5-point Likert scale (0 = Never to 4 = Always) and asked women if providers: were friendly/courteous; informed women about their care; used words/language that women could understand; gave women the opportunity to ask questions, and; gave reassurance during care. Open-ended questions solicited information on aspects that women liked most and least about their care and suggestions they had for improving care.

The four-week follow-up interview asked women about their general condition (i.e. good, fair, or poor) at the time of the interview and if any of the following occurred between discharge after delivery and four-weeks postpartum: symptoms of postpartum infection (i.e. pelvic and/or abdominal pain, foul-smelling discharge, fever); sought additional care for any reason; use of antibiotics; diagnosis of infection; hospitalization.

The primary outcome for this study was proportion of women self-reporting receipt of antibiotics for infection between hospital discharge after delivery and four weeks postpartum. Secondary outcomes included proportion of women treated for severe postpartum infection (defined as re-hospitalization and/or use of intravenous antibiotics for infection within four weeks postpartum) and level of postpartum pain experienced by women. Secondary outcomes also included women’s reported impressions on the quality of care received.

We estimated that approximately 43,200 vaginal deliveries would occur at study hospitals over the effectiveness trial and that 3.5% (n = 1512) of women would experience PPH and be eligible for the nested cohort study. We estimated that approximately half of women (n = 756) would deliver in the intervention period and 10% would have refractory PPH and receive UBT, leading to anticipated sample sizes of 75 women in the UBT cohort and 1437 women in the no-UBT cohort. Assuming a postpartum infection rate of 4% in the no-UBT group [28, 29], we estimated that this sample size would allow us to detect an approximate 2.5-fold increase in infection (one-sided alpha = 0.05, 80% power).

We used logistic regression with robust sandwich estimator to adjust standard errors for clustering by study facility [30] to compare UBT and no-UBT cohorts on baseline characteristics, including use of PPH interventions that could result in increased postpartum pain or increased risk of postpartum infection (i.e. use of urinary catheter, manual exploration/ removal of clots, suturing, bimanual compression, invasive surgery), use of pain management, and baseline use of antibiotics (during labor and/or delivery, during immediate postpartum period, and prescription of oral antibiotics at discharge). For outcome analysis of postpartum pain (reported in pre-discharge interviews), we reported the pain score as both a continuous outcome (reporting median score and interquartile range [IQR]) and as a categorical outcome using pain categories of mild (score 0–3), moderate (score 4–7) and high (score 8–10). Multinomial regression using an ordered outcome was used to calculate relative risk ratios (RRRs) and 95% CIs for pain category (mild being the base outcome) and self-reported condition at follow-up (categorized as good, fair, poor, good being the base outcome). Multivariable ordered multinomial regression of pain category with robust sandwich estimator to adjust standard errors for clustering by study facility was used to adjust for potential confounders of country, PPH interventions that may also result in increased pain (manual removal of placenta or manual evacuation of the uterus, bimanual compression), and use of pain management. Outcomes around women’s experiences with care (also reported during pre-discharge interviews) were analyzed for the whole cohort study population in line with our *a priori* analysis plan; we did not hypothesize that women’s overall experiences of care would differ significantly by UBT use and we were more interested in reporting on general trends among the whole population of women diagnosed with PPH, thus we did not compare these outcomes among the two cohorts. For closed-ended questions, overall proportions were reported. For open-ended questions, responses were reviewed, common themes identified, and responses were accordingly coded into categorical variables. In some cases, the follow-up visit happened earlier than 4 weeks postpartum so when analyzing outcomes measured at the 4-week follow-up interview, we restricted analysis to women who had the follow-up interview conducted at least 26 days after delivery. For analysis of postpartum receipt of antibiotics (reported in 4-week follow-up interviews), we conducted multivariable log-binomial regression with robust sandwich estimator to adjust standard errors for clustering by study facility to calculate RRs and 95% CIs after controlling for key covariates such as country, baseline use of antibiotics, and PPH interventions that may increase the risk of infection. All data analyses were conducted Stata 12 (StataCorp. 2011. *Stata Statistical Software*: *Release 12*. College Station, TX).

## Results

### Cohort characteristics

**Fig 1:**
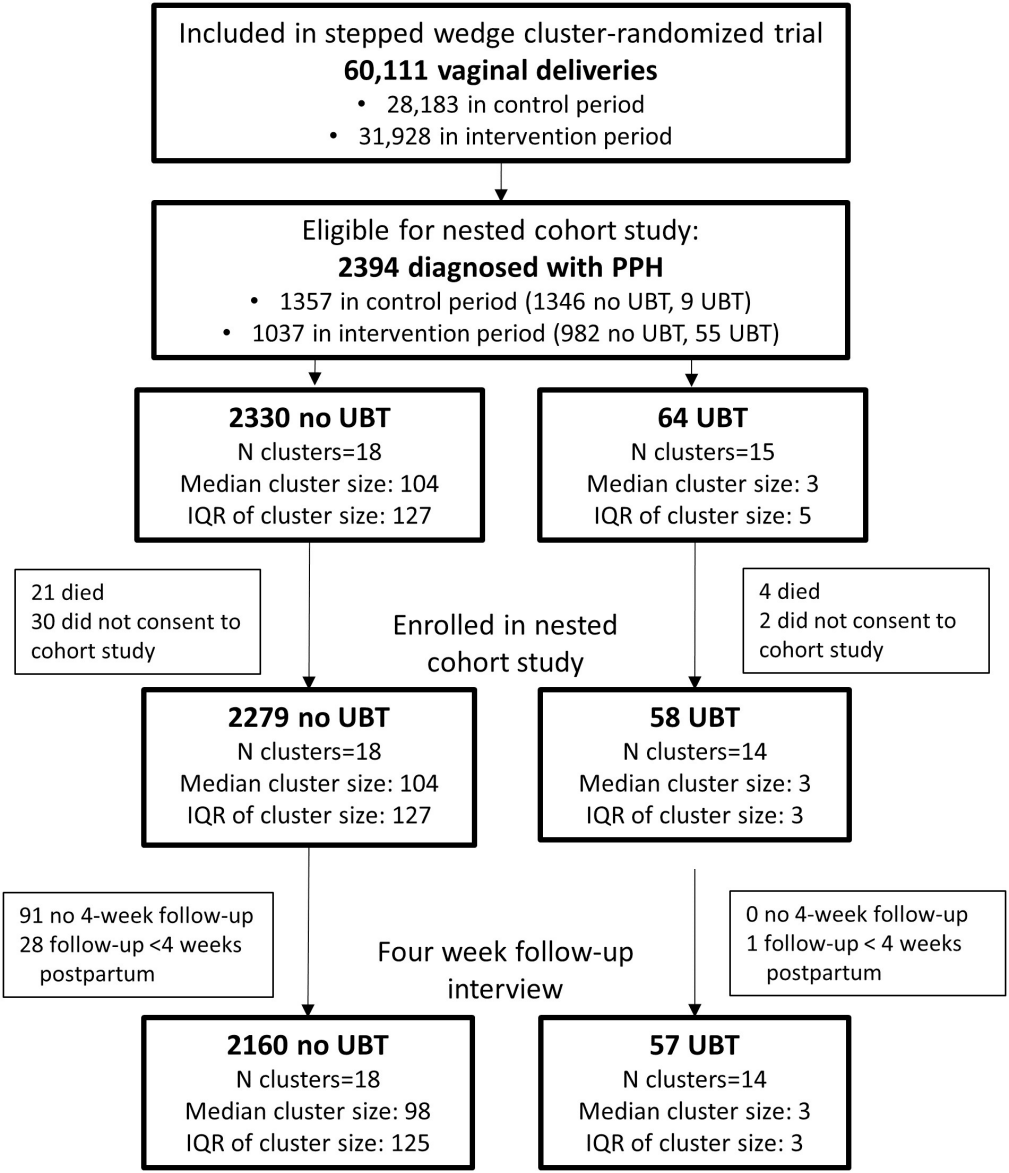
Enrollment and follow-up of cohort study nested in a stepped wedge, cluster-randomized trial of UBT introduction (i.e. effectiveness trial). Abbreviations: PPH = postpartum hemorrhage, UBT = uterine balloon tamponade.

Of the 60,111 vaginal deliveries that occurred during the effectiveness trial, 2394 (1357 and 1037 in the control and intervention periods of the effectiveness trial, respectively) were diagnosed with PPH and were eligible for the nested cohort study, including 64 women who had UBT and 2330 who did not receive UBT (Fig 1). Informed consent for participation in the cohort study and pre-discharge interviews were conducted for 58/64 (91%) and 2279/2330 (98%) women in the UBT and no-UBT groups, respectively, and this was the group for analysis of postpartum pain and women’s experiences with postpartum care. Follow-up interviews were completed at least four weeks postpartum for 57/58 (98.2%) women in the UBT group and for 2249/2279 (98.7%) in the no-UBT group, and this group was analyzed for outcomes measured at four week postpartum, including self-reported use of antibiotics postpartum and diagnosis of postpartum infection.

Analysis of baseline characteristics (Table 1) shows that the majority of women in both UBT and no-UBT groups were from Egypt, and that the UBT group had slightly higher proportions of women from Senegal (13.8% vs. 10.6) and Egypt (70.7% vs. 62.4%, p = 0.079). Women who received UBT were less likely to have PPH diagnosed at later timepoints after delivery, such as one to two hours (7.0% vs. 14.6%) or more than two hours after delivery (3.5% vs. 11.4%). Some PPH interventions were more common in the UBT cohort than in the no-UBT cohort, such as urinary catheterization (89.7% vs. 77.5%, p = 0.038) and invasive surgery (6.9% vs. 1.1%, p<0.001). Women in the UBT cohort were also more likely to receive antibiotics in the immediate postpartum period (90.0% vs. 48.3%, p<0.001).

**Table 1:** Background characteristics of women diagnosed with PPH and included in the nested cohort study.

	PPH, No-UBT group	PPH, UBT group	P value^a^
Total	2279	58	
**Baseline variables**			
Country			0.079
Uganda	616 (27.0%)	9 (15.5%)	
Egypt	1421 (62.4%)	41 (70.7%)	
Senegal	242 (10.6%)	8 (13.8%)	
Place of delivery			
Study facility	1960 (86.0%)	51 (87.9%)	0.700
Referred to facility for PPH after delivery elsewhere	319 (14.00%)	7 (12.1%)	
Live or stillbirth			
Live birth	2176 (96.0%)	54 (96.4%)	0.875
Stillbirth	91 (4.0%)	2 (3.6%)	
Unknown	12	2	
Time from birth to PPH diagnosis			0.014
0–14 min	516 (24.0%)	18 (31.6%)	
15–29 min	474 (22.0%)	17 (29.8%)	
30–59 min	601 (27.9%)	16 (28.1%)	
1–2 hrs	315 (14.6%)	4 (7.0%)	
>2 hrs	246 (11.4%)	3 (3.5%)	
Unknown	127	1	
**PPH interventions**			
Urinary catheter	1767 (77.5%)	52 (89.7%)	0.038
Manual exploration/removal of clots	1923 (84.4%)	54 (93.1%)	0.229
Suturing	898 (39.4%)	20 (34.5%)	0.506
Bimanual compression	1020 (44.8%)	33 (56.9%)	0.161
Invasive surgery	26 (1.1%)	4 (6.9%)	<0.001
**Pain management**^b^			
Received any pain management	679 (29.8%)	24 (41.4%)	0.366
General anesthesia	67 (2.9%)	2 (3.5%)	0.853
Regional anesthesia	13 (0.6%)	1 (1.7%)	0.313
Local anesthesia	129 (5.7%)	3 (5.2%)	0.918
Analgesic drugs	427 (18.7%)	16 (27.6%)	0.429
Sedative and/or ketamine	49 (2.2%)	2 (3.5%)	0.443
Unknown	1	0	
**Antibiotic use before or at discharge**			
Labor/delivery	565 (25.0%)	20 (34.5%)	0.225
Immediate postpartum	1093 (48.3%)	52 (90.0%)	<0.001
Discharged on oral antibiotics	1882 (83.1%)	48 (82.8%)	0.969
Unknown	16	0	
**Follow-up**			
Time to follow-up interview			0.652
<26 days	28 (1.3%)	1 (1.7%)	
26–35 days	1848 (85.4%)	51 (87.9%)	
≥35 days	312 (14.3%)	6 (10.3%)	
No follow-up interview	93	0	

^a^Likelihood ratio p values derived from logistic regression with clustered sandwich estimators.

^b^Pain medication not specified for 5 women all in no UBT group.

### Pre-discharge interviews: Pain after delivery and women’s experience with care

When asked before discharge about the highest level of pain experienced in the immediate postpartum period, 12/58 (20.7%) women in the UBT group and 478/2279 (20.9%) women in the no-UBT group reported that they didn’t know or could not recall. Among those who did answer, the median pain score was 7/10 among women in the UBT group (n = 46 IQR = 3) and 6/10 in the no-UBT group (n = 1801, IQR = 3). Mild, moderate, and high pain scores were reported by 5 (10.9%), 24 (52.2%) and 17 (37.0%) women in the UBT group, respectively, and by 383 (21.3%), 1060 (58.8%), and 358 (19.9%) women in the no-UBT group, respectively. In both cohorts, only 143/375 (38.1%) women who reported high pain scores received pain management and in the UBT cohort, 9/17 (52.9%) women who reported high pain scores received pain management. Multinomial regression revealed that women in the UBT group had similar odds of reporting a moderate pain score relative to a mild pain score (RRR = 1.73, 95% CI: 0.64–4.71) and had significantly greater odds of reporting a high pain score relative to a mild score (RRR = 3.64, 95% CI:1.30–10.16). This relationship remained statistically significant after adjusting for co-variates such as other painful procedures and use of pain management, though it was not statistically significant after adjusting for country (RRR = 2.68, 95% CI:0.79–9.09). When asked specifically about pain at time of balloon insertion, 23/58 (39.7%) women who received UBT said they did not know or could not recall and among the 35 who did answer, the median pain score was 8 (IQR = 3), including 6 (17.1%) women reporting a mild pain score, 11 (31.4%) reporting a moderate pain score, and 18 (51.4%) reporting a high pain score.

**Fig 2:**
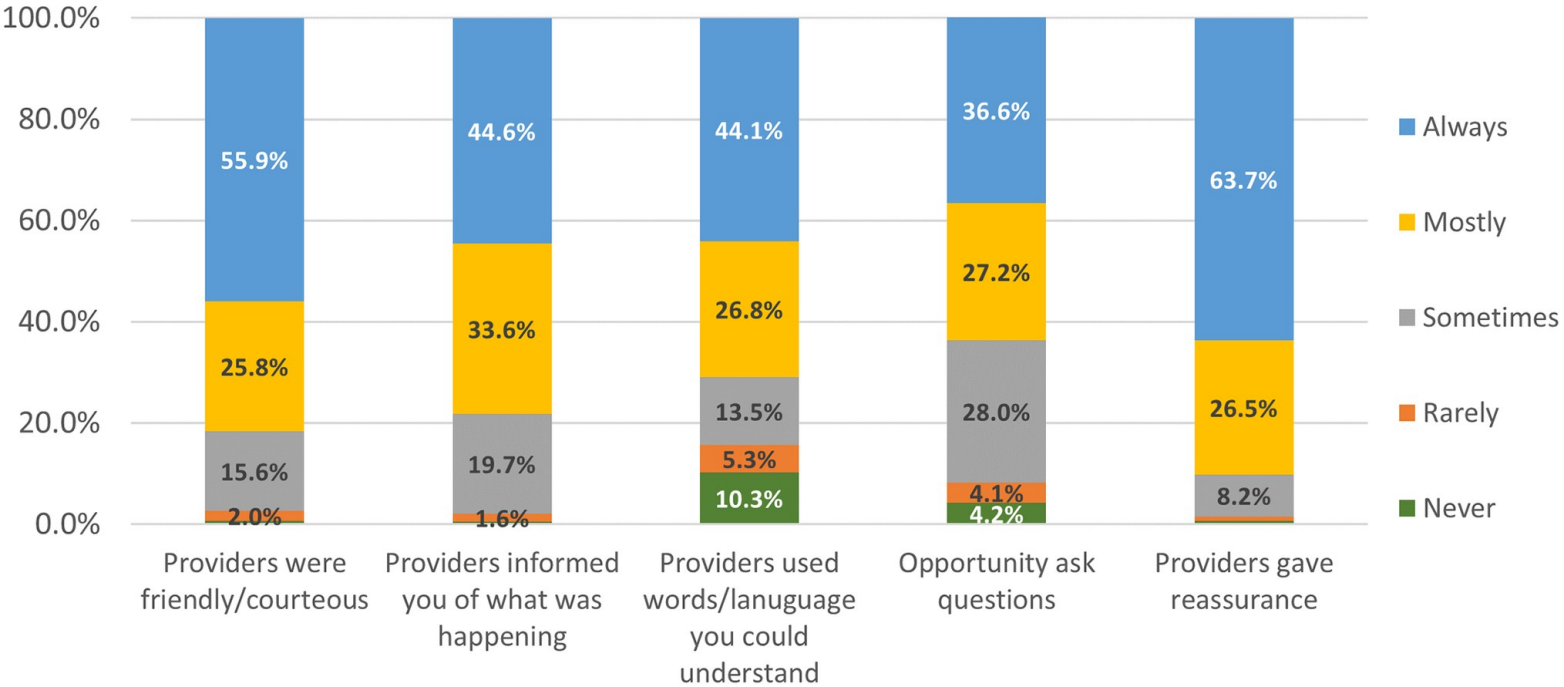
Self-reported experiences with care among 2313 women diagnosed with PPH at secondary level health facilities in Uganda, Egypt, and Senegal.

Information on women’s experiences with care are reported for the combined UBT and no-UBT cohort. Most women had good impressions of their overall care (Fig 2). For example, women reported that providers mostly or always: were friendly and courteous (81.7%); informed them of what was happening (78.3%), or; gave them reassurance (90.2%). Notably, 29.1% of women reported that providers sometimes, rarely, or never used language they could understand, and 36.3% said they were sometimes, rarely, or never given the opportunity to ask questions. Several women (412/2329, 17.7%) reported calling out for help during their hospital stay. The top reasons women called for help were because they or their companions noted excessive bleeding (170/412, 41.3%) or they were in pain (105/412, 25.5%, data not shown).

Most women reported being satisfied with care (1934/2323, 83.2%) and the aspects they liked most were good quality of care (44%), fast and efficient care (26%), kind and supportive staff (24%), and skilled and committed providers (12%, Table 2). Substantial proportions of women declined to answer the questions of what they liked least about their care (827/2339, 35.4%) and what suggestions they had for improving care (1068/2339, 45.7%). Of 1511 women who did report what they liked least, the most common responses were: medication was unavailable and/or had to buy medication from outside the facility (n = 503, 33.3%), medical supplies were unavailable and/or had to buy supplies from outside the facility (n = 246, 16.3%), facilities were lacking or unclean (n = 83, 5.5%), had painful procedure/untreated pain (n = 49, 3.2%), and staff were rude, abusive or neglectful (n = 73, 4.8%). Among 1271 women who provided suggestions on how to improve care for women who have PPH, common responses were to address problems around availability of medications (n = 343, 27.0%), medical supplies (n = 178, 14.0%), and blood (n = 58, 4.7%).

**Table 2:** Self-reported experiences with care among 2339 women diagnosed with PPH before discharge after delivery.

	Total, N = 2337
**Satisfaction with care received at the facility**	**N = 2323**
Satisfied	1934 (83.2%)
More or less satisfied	376 (16.2%)
Not satisfied	14 (0.6%)
**How likely to recommend this facility to someone else**	**N = 2332**
Very likely to recommend	1969 (84.4%)
Somewhat likely to recommend	335 (14.4%)
Not likely to recommend	28 (1.2%)
**Would return to this facility for a future pregnancy**	**N = 2316**
No	49 (2.1%)
Yes	2019 (87.2%)
Not applicable, not planning another pregnancy	248 (10.7%)
**Aspects liked most about the care received at the facility**	**N = 2023**
Good care/treatment/service, good quality care	897 (44.3%)
Fast/rapid/efficient care	532 (26.3%)
Friendly/kind/welcoming/empathetic/supportive staff	476 (23.5%)
Skilled/hardworking/committed providers	236 (11.7%)
Presence of skilled medical staff (doctors/midwives/ nurses)	127 (6.3%)
Saved woman’s life/stopped the bleeding	250 (12.4%)
**Aspects liked least about care at this facility**	**N = 1510**^a^
Disliked nothing	579 (38.3%)
Medication not available/ bought medication outside	503 (33.3%)
Medical supplies not available/ bought supplies outside	246 (16.3%)
Facilities were lacking or unclean (e.g. no sheets, not enough beds, dirty toilets)	83 (5.5%)
Severe pain/untreated pain/painful procedure	49 (3.2%)
Staff were rude or abusive/treated badly by staff/unresponsive or neglectful care	73 (4.8%)
Blood/blood type not available	22 (1.5%)
Other	169 (11.2%)
**Suggestions on how to improve the care at this hospital for women who have PPH**	**N = 1269**^b^
No suggestion/suggest to continue in same manner	476 (37.5%)
Ensure medications are available/give medication for bleeding	343 (27.0%)
Ensure necessary medical supplies available	177 (14.0%)
General improvements in care (e.g. better monitoring, more timely response, overall quality)	62 (4.9%)
Ensure blood/all blood types available	58 (4.6%)
Improve hospital conditions (e.g. more beds, provide sheets, cleanliness)	78 (6.1%)
Educate women to come to hospital for delivery/ self-care during prenatal period	47 (3.7%)
Stop painful procedures/provide pain management	40 (3.2%)
Improvements in staffing (e.g. increase number of providers, change health staff and/or improve attitude of staff)	60 (4.7%)
Other improvements	65 (5.1%)

^a^No answer from 827/2337 (35.4%) women (794 no UBT group, 33 UBT group),

^b^No answer from 1068/2337 (45.7%) women (1034 no UBT group, 34 UBT group).

### Follow-up interviews at 4-weeks postpartum: Postpartum infection

In follow-up interviews, 9/57 (15.8%) and 253/2160 (11.7%) women in UBT and no-UBT groups sought additional care during the four weeks after discharge (RR = 1.35, 95% CI: 1.01–1.80). Though UBT and no-UBT groups were similar (RR = 0.83, 95% CI: 0.53–1.30) regarding self-reported symptoms during the four-week follow-up period (including fever, abdominal pain/cramps, heavy bleeding, or foul-smelling vaginal discharge), the groups differed slightly regarding women’s self-reported general condition at 4 weeks postpartum, with a good, fair, or poor condition reported by 46 (80.7%), 9 (15.8%), and 2 (3.5%) of women in the UBT group, respectively, and by 1777 (82.3%), 372 (17.2%), and 11 (0.5%) women in the no-UBT group (RRR for outcome of poor vs. good = 6.97, 95% CI: 2.82–17.27). Similar proportions of women in the UBT cohort (3/53, 5.7%) and no-UBT cohort (99/2141, 4.6%) reported the primary outcome of receiving antibiotics during the four-week follow-up period (RR = 1.22, 95% CI: 0.45–3.35, Table 3). This finding was unchanged after adjusting for country, use of antibiotics, and other interventions that could increase infection risk (i.e. manual exploration, bimanual compression, surgery). Few women in both groups reported receiving treatment for severe postpartum infection (0/53 in UBT group, 16/2141 [0.8%] in no-UBT group, p = 1.000). No women in the UBT group and 50/2114 (2.4%) women in the no-UBT group reported receiving a diagnosis of infection within the follow-up period.

**Table 3:** Self-reported postpartum infection during the 4-week postpartum follow-up period.

	PPH, No-UBT group	PPH, UBT group	Risk ratio (95% CI) ^a^	P value^a^
Total, N	2160	57		
**Primary outcome**	**N = 2141**	**N = 53**		
Woman reported receiving antibiotics during 4 weeks postpartum^b^	99 (4.6%)	3 (5.7%)	1.22 (0.45–3.35)	0.694
**Secondary outcomes**				
Treatment for severe postpartum infection during 4 weeks postpartum^c^^,^ ^d^	N = 2141	N = 53		
	16 (0.8%)	0	-	-
Diagnosed with postpartum infection during 4 weeks postpartum^d^^,^^e^	N = 2114	N = 55		
	50 (2.4%)	0	-	-
Type of Infection	N = 50	-		
Endometritis	9	-	-	-
Urinary tract infection	23	-	-	-
Other^f^	18	-	-	-

^a^95% confidence intervals (CIs) and p values derived from log-binomial regression with clustered sandwich estimators.

^b^Excludes women who said they didn’t know or didn’t remember if they had received antibiotics during 4 weeks postpartum (n = 19 in no UBT group, n = 4 in UBT group).

^c^Defined as women who were hospitalized to treat postpartum infection (n = 1) or received intravenous antibiotics (n = 11) or both (n = 4) for postpartum infection within 4 weeks after delivery, excludes women who said they didn’t know or didn’t remember (n = 19 in no UBT group, n = 4 in UBT group).

^d^Rates are too small to allow valid confidence interval estimation and significance testing.

^e^Women reported whether they were aware of a diagnosis of infection within 4 weeks postpartum, excludes women who said they didn’t know or didn’t remember (n = 46 in no UBT group, n = 2 in UBT group).

^f^Includes women who report diagnosis of mastitis (n = 4), infection of sutures (n = 2), yeast/fungal infection (n = 2), puerperal sepsis (n = 1), unspecified infection during pregnancy that persisted postpartum (n = 1), unspecified sexually transmitted infection (n = 1), pneumonia (n = 1), acute pelvic inflammatory disease (n = 1), unspecified vaginal infection (n = 1), “bad hygiene” (n = 1), “fever” (n = 1), pelvic bleeding on and off (n = 1), infected wound (n = 1).

## Discussion

One of the concerns around expanding use of UBT in LMICs is the possibility of exposing more women to an increased risk of infection. This prospective cohort study of women diagnosed with PPH shows that use of UBT did not result in an increased risk of postpartum infection based on a variety of self-reported measures, including use of postpartum antibiotics, treatment of severe infection, or diagnosis of infection. These findings corroborate results from previous published case series showing low rates of infection among women receiving UBT [8, 31–33]. However, our findings do not confirm those documented in a secondary analysis of the World Maternal Antifibrinolytic (WOMAN) trial that included 20,000 women treated for PPH that showed that use of UBT was associated with a an increased risk of sepsis [18]. The secondary analysis paper also showed that women who had laparotomy or hysterectomy also had an increased risk of sepsis, and it may be difficult to tease out the individual contribution of UBT use to sepsis, since women who received UBT may have been more likely to receive interventions such as invasive surgery that may have also increased their risk of sepsis. Further, the WOMAN trial did not collect information on use of antibiotics; thus, it is unknown how many women in the study population received antibiotics and the authors were not able to control for antibiotic use in the analysis. In our study, the abundant use of prophylactic antibiotics may have contributed to low rates of postpartum infection in the study population overall. While our multivariable analysis showed that controlling for antibiotic use did not change our main findings, it is unclear if our findings are generalizable to settings with less antibiotic use.

Our study is novel in that it reports information on pain associated with UBT use, an element of acceptability that has been under-reported in other published studies of UBT. Women with PPH who received UBT were more likely to report a high pain score compared to women who did not have UBT. This association of UBT use and greater pain is not statistically significant when controlling for country, which could reflect that some countries had higher rates of UBT use and cultural variations that influence how individuals experience and report pain [34, 35]. When asked specifically about pain at the time of balloon insertion, 51% of women who received UBT reported pain scores of eight or above on a 0–10 scale, suggesting that UBT may be the direct cause of increased pain for some women. Notably, a fairly large proportion of women answered “don’t know” when asked about postpartum pain, which may reflect some women’s difficulty interpreting their pain on a 0–10 scale. The proportion of women who responded “don’t know” about pain was identical in the UBT and no-UBT group, so this probably did not impact the finding of higher pain scores in the UBT group. Findings of higher pain associated with UBT should not necessarily deter UBT use for appropriate cases where it may be beneficial (and potentially avert surgery and greater pain, particularly if appropriate pain management is not available). Rather, these findings are relevant for helping shape clinical protocols around UBT use regarding the potential need for appropriate pain management when UBT is used. The study training advised providers to offer pain medication as needed to women who received UBT; however, pain medication was not given to 41.4% of women who received UBT, including 9/17 (47.1%) women who had UBT and reported high pain scores. Future research may help elucidate whether provision of pain medication to all women who receive UBT would be advantageous and what pain medications may be most appropriate for this procedure.

Information on women’s overall experiences suggest that most were categorically satisfied with their PPH care, although open-ended responses revealed important considerations for implementation of PPH interventions like UBT. For example, similar to previous studies [20, 25], many women remarked that aspects they liked least about their care were that medications and medical supplies were not available and that they or their families had to procure these from outside the facility. Such factors could delay or even preclude (if women are unable to pay) the provision of urgently needed interventions.

Poor quality care and mistreatment of women were other negative aspects mentioned by women. Some reported mistreatment or neglect by staff, which could result in a hesitancy of women to seek out the help of providers if they sense a problem and, in turn, lead to delays in receiving needed interventions. Prior studies have shown that mistreatment of women during childbirth is not uncommon in LMICs [24, 25], and such mistreatment can negatively impact health-seeking behavior. Women also said they disliked painful procedures done postpartum, which indicates that pain management may not have been adequate. Indeed, offering pain management is a component of high-quality maternity care [36].

Our study has some limitations. Though we enrolled a larger number of women diagnosed with PPH than anticipated in sample size calculations, fewer women than expected received UBT for PPH. This may have compromised our ability to estimate accurately the incidence of postpartum infection in women who received UBT and to detect a difference in infection rates. Our initial sample size and minimum detectable difference estimations did not account for clustering by study site because UBT was eventually introduced at all study sites; however; UBT use and postpartum infection rates could be impacted by study site characteristics, and we did account for study site in the analysis. Due to these potential clustering effects, we likely had less statistical power to detect a difference in postpartum infection rates (as measured by self-reported post-partum antibiotic use); yet, the low rates of infection-related outcomes in both the UBT and no-UBT groups suggest that any difference between the two groups, even if statistically significant, is likely to be small. Second, infection outcomes were all self-reported by women four weeks after delivery and could have been subject to recall bias; for example, women may have incorrectly identified medications they received as antibiotics. In addition, antibiotics may have been given after discharge during the postpartum period for prophylactic reasons, such as to women with prolonged lochia. Such misclassification is likely non-differential according to UBT use, thus this likely did not impact our comparative analysis of infection rates. Findings around self-reported pain and women’s experience of care may have been impacted by women’s reluctance to report negative experiences to interviewers, who were often hospital personnel. Finally, the UBT and no-UBT cohorts differed on several factors. For example, the UBT cohort likely had a higher proportion of women with refractory PPH based on the indications for UBT use and the significantly higher rate of invasive procedures performed among women who received UBT. The use of surgery and other invasive interventions could lead to an increased risk of postpartum infection and increased levels of pain that were unrelated to UBT use. We attempted to account for this by controlling for surgery and other procedures such as manual exploration and bimanual compression which may cause more pain and may also be more common among women with refractory PPH. However, it is still possible that other factors associated with refractory PPH caused the higher pain scores. Our study is strengthened by the prospective design, inclusion of a large number of women diagnosed with PPH, and a high follow-up rate at 4-weeks postpartum.

## Conclusions

In conclusion, this study conducted in three LMICs shows that UBT does not result in an increased risk of postpartum infection. In addition, this study shows that the need for pain management and reassurance should be anticipated when UBT is used. Further, when implementing an intervention like UBT in LMICs, it is important to consider the overarching challenges to provision of high quality PPH care, such as problems in maintaining adequate stocks of basic, first-line interventions. The introduction of a new supply-dependent intervention may result in additional supply problems and pass more costs on to women. Future implementation research on PPH interventions would help elucidate how interventions can best be introduced into already overburdened health systems.
